# Methods for modelling composite indices of access to healthcare facilities: a systematic literature review

**DOI:** 10.1186/s12963-025-00432-7

**Published:** 2025-11-21

**Authors:** Moses M. Musau, Ann Njogu, Alex Maina, Robert W. Snow, Lenka Beňová, Emelda A. Okiro, Catherine Linard, Peter M. Macharia

**Affiliations:** 1 Kenya Medical Research Institute (KEMRI) - Wellcome Trust Research Programme, Nairobi, Kenya; 2 Institute of Life, Earth, and Environment (ILEE), University of Namur, Namur, Belgium; 3 Department of Public health, Institute of Tropical Medicine Antwerp, Antwerp, Belgium; 4 Centre for Tropical Medicine and Global Health, Nuffield Department of Clinical Medicine, University of Oxford, Oxford, UK; 5 Faculty of Epidemiology and Population Health, London School of Hygiene and Tropical Medicine, London, UK; 6 Namur Research Institute for Life Sciences (NARILIS), University of Namur, Namur, Belgium

**Keywords:** Healthcare access, Composite index, Multidimensional access, Affordability, Availability, Accessibility, Accommodation, Acceptability

## Abstract

**Background:**

Access to quality healthcare services is key to achieving Universal Health Coverage (UHC). The multidimensional nature of access (availability, accessibility, accommodation, affordability and acceptability) makes it challenging to quantify the level of access. Current approaches focus predominantly on single dimensions, limiting the comprehensive monitoring and evaluation of access to healthcare facilities. Here, we conduct a systematic literature review on the methodological approaches and data used to construct multidimensional composite indices of healthcare facility access, globally.

**Methods:**

We undertook a literature search in eight databases including EBSCOhost (CINAHL), Google Scholar, Ovid (Embase and MEDLINE), PubMed, Scopus, Web of Science and Web of Science (MEDLINE) adhering to the Preferred Reporting Items for Systematic Reviews (PRISMA) guidelines. Studies that incorporated multiple dimensions of access to healthcare facilities to construct a composite index were considered and quality assessment performed. Methodological approaches to measuring access and their supporting conceptual frameworks were synthesised using descriptive summaries and thematic analysis.

**Results:**

Out of 4,291 articles retrieved,19 met inclusion criteria with an average quality score of 19.6 out of 26. Most of the studies (68%) were conducted in 2021–2024, mainly in India (53%) or USA (16%); none in Africa. The composite indices of access combined two (32%), three (42%), four (5%) or all five dimensions (21%), with affordability (84%) being the most frequent dimension. There was significant heterogeneity on the definition, data (survey-based or retrospective) and representation of indicators. There were four weighting techniques ranging from simple (equal weighting) to complex approaches (Principal Component Analysis and Analytical Hierarchy Process). Studies used four different approaches to combine indicators; arithmetic mean (ten studies), summation (six studies), Adjusted Mazziotta-Pareto Index (two studies) and geometric mean (one study). Only 63% validated their output.

**Conclusions:**

There is diversity in the approaches used for multidimensional assessment of access to healthcare facilities. To ensure robust, context-specific and more comprehensive composite indices, the use of clearly defined frameworks, dimension weights that reflect context-specific access barriers and penalised aggregation methods will be required.

**Supplementary Information:**

The online version contains supplementary material available at 10.1186/s12963-025-00432-7.

## Introduction

Effective healthcare delivery depends on equitable access to healthcare services for the population as enshrined in the Alma-Ata Declaration in 1978 [1]. This declaration recognised that health was not only due to the physical presence of hospitals and health staff but also due to factors associated with access to services; education, social and economic status and individual choices [1, 2]. Under the current Sustainable Development Goal (SDG) 3, enhancing universal access to quality healthcare services is fundamental towards ensuring healthy lives and promoting well-being for all [2, 3].

The concept of access to healthcare services is complex, encompassing various dimensions [4–6]. According to Penchansky & Thomas [4], there are five distinct taxonomies of access to healthcare services: availability, accessibility, accommodation, affordability and acceptability. Availability relates to the volume and match of the existing healthcare services and the healthcare needs of the population in terms of both quality and quantity; accessibility is used to define the physical separation in the location of the healthcare service providers and the population and the involved costs such as distance and travel time; accommodation assesses the congruence between the organisational structure of the healthcare services provision including operational hours, appointment procedures in relation to the expectations of the served population; affordability relates to the population’s financial ability to pay for the healthcare services rendered; and acceptability examines the alignment between the population’s expectations and actual delivery of healthcare services based on socio-demographics factors such as economic status, religion, age and ethnicity.

Levesque et al. [6] incorporates a patient-centred approach and defines access as a process of seeking care, focused on the stages a patient goes through to receive the needed care, and proposes five dimensions; (a) approachability, (b) acceptability, (c) availability and accommodation; (d) affordability; and (e) appropriateness. Levesque et al. [6] retains four dimensions and groups these into three by combining availability and accommodation; while introducing the concept of approachability - the ability of a population with a healthcare need to identify the existence of the healthcare services that can satiate their healthcare need - and appropriateness, the agreement of the healthcare services rendered with the healthcare demands of a population [6]. The population’s awareness of existing services and treatments from exposure to information via media or outreach programs is a key factor in determining approachability. In contrast, the correctness of the treatment rendered including the level of technical and interpersonal expertise, characterises the appropriateness dimension [6].

While these two frameworks of access are related, henceforth, our analysis adopts the Penchansky & Thomas framework [4] as its focus on the healthcare system’s ability to deliver care relative to the demand and characteristics of the population served aligns with facility-centric assessments as opposed to the framework by Levesque et al. [6] whose focus is on patient journeys from need recognition to appropriate care.

To comprehensively evaluate and understand access to healthcare services, it is necessary to simultaneously consider and integrate the different dimensions of access to define a more comprehensive index of multi-dimensional access to healthcare services [4, 6]. However, creating and consequently interpreting a more comprehensive index that acknowledges the multi-dimensionality of access is not trivial; data availability issues, aggregation methods that capture the interrelatedness complexities and the overall low demand of the composite indices. As a result, there is limited adoption of multidimensional approaches for quantifying access to healthcare services across existing literature, with predominant focus on the individual access dimensions such as spatial accessibility [7–10], affordability [11], availability and demand [12, 13], acceptability [14] and accommodation [15]. While these efforts have allowed a better understanding of the individual dimensions of access to healthcare services, they are limited because each dimension of access to healthcare services is not autonomous, instead, these dimensions are largely interrelated [4–6, 16–18]. Additionally, the existing multidimensional approaches are not well documented and a framework for best practices is lacking.

The only existing review in this space conducted a systematic literature review focusing on methods used to create a composite index by combining only two particular dimensions, accessibility and availability (altogether known as spatial accessibility) through a specific approach- gravity models [19]. However, a methodological systematic review on existing literature that constructs a composite index of access to healthcare services combining all the five dimensions of access or other combination pairs across the access dimensions (other than those described by Stacherl & Sauzet [19]) is lacking.

The collation and synthesis of such literature on composite access index is important to identify potential strengths and weaknesses of existing methods used in healthcare literature and proposing a methodological framework for comprehensively quantifying access to healthcare factoring in all the dimensions of access.

Here, we conduct a systematic literature review on existing techniques used for defining composite metrics for quantifying access to physical points of healthcare service provision (here referred to as healthcare facilities). Through the Penchansky & Thomas framework [4] lens, we aim to review the methods and datasets that have been used in literature to combine two or more dimensions of access to healthcare facilities to construct a healthcare access composite index. Further, to identify the limitations/opportunities of the methods and datasets used to define the composite index.

## Methods

### Search strategy and selection criteria

The systematic review was conducted adhering to the Preferred Reporting Items for Systematic Reviews (PRISMA) guidelines [20, 21] and the study protocol was registered on Open Science Framework [22]. The search strategy included relevant search terms, Boolean terms and truncations to allow for a comprehensive search (Supplementary file, Search strategy) and was executed on 14/01/2025. The databases for the literature search were EBSCOhost CINAHL, Google Scholar, Ovid (EMBASE and MEDLINE), PubMed, Scopus, Web of Science (WoS) and WoS (MEDLINE). For Google Scholar database, the top 200 hits were selected to capture the most relevant literature from the database platform. The search results from these electronic databases were then exported to Rayyan [23] (Rayyan Systems Inc., 2016) for deduplication and screening.

Each record was reviewed based on the condition/content and population (CoCoPop) criteria. There were no restrictions on date of publication or geographical scope. Specifically, the inclusion criteria were (i) articles that defined access to healthcare facilities (condition) using more than one dimension of access (context), (ii) articles reporting data on the general population (articles on incarcerated or homeless populations were excluded) and articles published in English. We excluded studies that (i) reported access to non-physical points of care such as telemedicine and community healthcare workers as their logistical, policy and equity mechanisms differ from patient-initiated care seeking at physical healthcare facilities; (ii) articles on single dimension of access; (iii) articles that did not construct a composite index of access, iv) articles not published in English and v) conference abstracts, reports and literature review articles. In addition, given the recent comprehensive review by Stacherl & Sauzet [19] on gravity models for combining the availability and accessibility dimensions, we excluded articles that solely used these gravity models to combine availability and accessibility dimensions.

### Data extraction and synthesis

Deduplication was done in the Rayyan software tool and MMM and AN independently undertook the study selection process by first performing title/abstract screening and then conducting full-text screening of the selected articles. Disagreements in the inclusion/exclusion of articles in both screening stages were resolved by a consensus between the two reviewers and by PMM as an arbitrator. Gwet’s AC1 statistic was used to assess the inter-rater agreement. Additional articles were retrieved from snowballing the references of the included articles during full-text review and data extraction processes. The data extracted from the included articles (i) full article reference; (ii) access to healthcare facilities, dimensions and datasets used; (3) models utilised to construct composite access index and the validation techniques; (4) additional datasets/covariates; and (5) limitations and recommendations (Supplemental Table 1). For each article, synthesis of the dimension definitions, datasets and indicators used and the methods for constructing a composite access index were synthesised using descriptive summaries and thematic analysis.

### Quality assessment

The quality assessment tool from Harris et al. [24] (Supplemental Table 2) was adopted. This tool assesses article quality in three sections: (1) screening – assesses clarity and comprehensiveness of study objectives, study setting, population and outcome measures, (2) model validity – assesses model appropriateness, description and validation and (3) results and conclusions – assesses the comprehensiveness and interpretation of the results. Each item is scored between 0 and 26 and grouped as 0 (poor), 1 (moderate) or 2 (good) quality. The total score for each study was interpreted as ‘low’ if < 14, ‘medium’ if 14–18, ‘high’ if 19–22 and ‘very high’ if >22.

## Results

### Article search, inclusion/exclusion and quality assessment

**Fig. 1:**
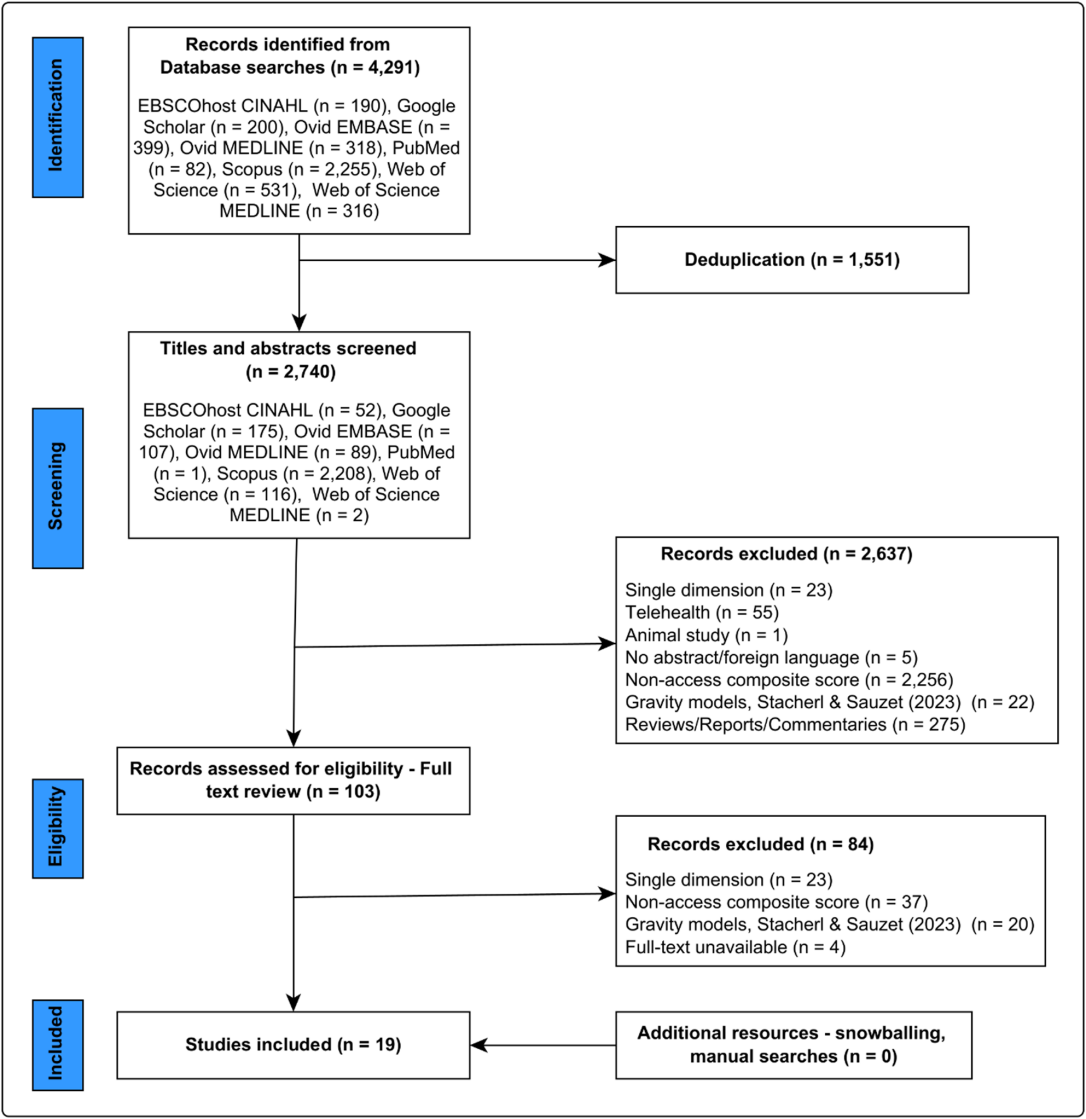
PRISMA flow chart on study identification, screening and selection for the systematic review

A total of 4,291 articles were retrieved from the initial search across the eight electronic databases – snowballing did not identify any new studies that met our inclusion criteria. After deduplication, 1,551 articles were excluded. Of the remaining 2,740 articles that underwent title and abstract screening, 2,637 did not meet the inclusion criteria (Fig. 1). A full-text review was conducted for 103 articles where a total of 19 studies were included for data extraction and synthesis (Fig. 1). There was 96.1% (Gwet’s AC1 = 0.958) agreement between MMM and AN on article inclusion/exclusion. Quality assessment was performed for all 19 included studies (max 26); the mean score was high, 19, ranging from 12 to 25 (Supplemental Table 3).

### Characteristics of included studies

Of the 19 included studies, ten (53%) were from India, three (16%) from the USA while Argentina, Ecuador, England, Italy, Oman and South Korea had one (5.3%) study each (Table 1). Notably, only one study was published before the year 2010, with 13 (68.4%) of the studies being published between 2021 and 2024. The majority of studies (11, 58%), focused on access to general healthcare services while the rest focused on specific services/conditions including asthma, breast cancer, Coronavirus disease 2019 (COVID-19), dental care, diabetes, Human Immunodeficiency Virus (HIV), reproductive healthcare and surgery. For general healthcare services, access was defined to general healthcare facilities. In contrast, for condition/service-specific studies, access was defined to healthcare facilities with the capacity to provide relevant services.

The studies utilised either surveys (of patient or households) (8, 42%), or secondary data sources (11, 58%), to obtain the datasets for the different access dimensions (Table 1). Survey-based studies involved administering Likert-scaled questionnaires focused on access dimensions to patients or household heads and the analysis was conducted at either individual level, (7, 37%) or the household level (1, 5.3%). In contrast, retrospective/secondary analysis studies utilised and analysed secondary data at different administrative boundary level.

**Table 1:** Study characteristics of 19 included studies on access to health care

Study characteristics	*N* = 19
**Country**	
India	10 (53%)
USA	3 (16%)
Argentina	1 (5.3%)
Ecuador	1 (5.3%)
England	1 (5.3%)
Italy	1 (5.3%)
Oman	1 (5.3%)
South Korea	1 (5.3%)
**Publication year**	
1998	1 (5.3%)
2010–2020	5 (26.3%)
2021–2024	13 (68.4%)
**Study outcome (type of healthcare)**	
General healthcare	11 (58%)
Asthma	1 (5.3%)
Breast cancer	1 (5.3%)
COVID-19	1 (5.3%)
Dental care	1 (5.3%)
Diabetes	1 (5.3%)
HIV	1 (5.3%)
Reproductive health care	1 (5.3%)
Surgery	1 (5.3%)
**Data Collection**	
Retrospective/Secondary data analysis	11 (58%)
Survey	8 (42%)
**Spatial resolution of access index**	
Administrative boundaries	11 (58%)
Individual	7 (37%)
Household	1 (5.3%)

COVID-19 - Coronavirus disease 2019; HIV - Human Immunodeficiency Virus

Overall, affordability was the most included dimension across the studies, (*n* = 16), while accessibility (*n* = 9) was the least factored in dimension (Table 2). Six studies combined two dimensions of access, with availability and affordability being the most frequent combination (*n* = 3). Eight studies combined three dimensions of access, one study combined four dimensions, and five studies combined all five dimensions of access (Table 2). Notably, four out of these five studies were done between 2020 and 2024.

**Table 2:** A summary of access dimensions described in the included studies

	*n*	References
**Distribution of dimensions across studies**		
Affordability	16	[25–30], 32– [38, 40, 42, 43]
Availability	14	[25–32], 35– [38, 40, 41]
Acceptability	11	[28, 32–36, 39–43]
Accommodation	10	[28, 30], 32– [34, 36, 39, 40, 42]
Accessibility	9	[25, 27, 28], 31– [33, 36, 40, 41]
**Two dimensions (n = 6)**		
Availability, Affordability	3	[26, 29, 37]
Accessibility, Availability	1	[31]
Accommodation, Acceptability	1	[39]
Affordability, Acceptability	1	[43]
**Three dimensions (n = 8)**		
Accessibility, Availability, Affordability	2	[25, 27]
Affordability, Accommodation, Acceptability	2	[34, 42]
Availability, Affordability, Accommodation	2	[30, 38]
Accessibility, Availability, Acceptability	1	[41]
Availability, Affordability, Acceptability	1	[35]
**Four dimensions (n = 1)**		
Accessibility, Affordability, Accommodation, Acceptability	1	[33]
**All the dimensions (n = 4)**		
Accessibility, Availability, Affordability, Accommodation, Acceptability	4	[28, 32, 36, 40]

### Dimensions of access and their definitions

Each study defined and selected indicator datasets for the access dimensions in line with their study context and data sources/collection methods. We synthesised the definitions and datasets used across the included studies cutting across both subjective measures from surveys and objective metrics from secondary analysis studies.

#### Geographic accessibility

The definition of geographic accessibility across the studies emphasised the spatial relationship between the location of healthcare facilities and that of the population, with a focus on travel impedance (distance and travel time) and barriers to transportation (Supplemental Table 4). Both subjective and objective datasets were used to define geographic accessibility. For example, survey-based studies utilised subjective survey responses from patients/households about the perceived distance and/or travel time from their homes to the healthcare facilities. In addition, behavioural indicator questions on accessibility, such as a reason for not seeking care based on the ease and costs of transportation involved, were included in survey-based studies to measure accessibility. On the other hand, objective measures included straight-line distances to healthcare facilities, Thiesen polygons and modelled travel times. These were often expressed as a proportion of the population or villages within a pre-defined travel time threshold. Overall, the datasets used to derive geographic accessibility metrics were road network, topography, travel speeds, ambulatory services, urbanisation and healthcare facilities locations (Supplemental Table 4).

#### Availability

The availability dimension captured the adequacy in supply of healthcare resources relative to the demands of the population (Supplemental Table 5). The approaches used to measure availability were highly variable and cut across institutional, i.e. the presence of healthcare facilities, human resources, services, and material/equipment aspects. Specific indicators included the number and spatial distribution of healthcare facilities, staffing to population ratios, beds per unit of population and availability of prescribed medications. Outcome-specific studies included service-specific and cadre-specific availability measures, for example, the study by Debnath et al. [25] focused on reproductive healthcare services included the availability of antenatal care services, normal delivery services, operation theatre, 24 × 7 services, and ratios of medical officers at the healthcare facilities (Supplemental Table 5). Several studies defined availability based on service capacity relative to existing industry benchmarks such as the proportion of available medicines in a healthcare facility from the list of the Indian public standards [26, 27]. On the other hand, subjective measures mainly from surveys included waiting times, perceived availability of medical professionals, medicines and/or tests when needed, and the ease of obtaining the prescribed medicine and/or medical treatment [28, 36, 41]. One of the studies included the aspect of staff qualification in their assessment of availability [28].

#### Affordability

The population’s willingness and ability to financially cater for healthcare services was used across the included studies to characterise the affordability dimension (Supplemental Table 6). The dimension was assessed using metrics for direct expenditure, financial protection and socioeconomic measures [25–30, 32–38, 40, 42, 43]. Direct expenditure indicators included out-of-pocket costs, household health spending and catastrophic health expenditure thresholds; financial protection indicators included insurance coverage and enrolment into other financial protection schemes; and material living standards and household income levels were used as socioeconomic indicators (Supplemental Table 6). Healthcare utilisation metrics such as antenatal care, facility delivery and immunisation were also used to measure affordability in some of the studies [29, 30]. Survey-based studies focused on the ease of financially catering for healthcare services and whether affordability was a significant factor in choosing to seek or not to seek care. Specifically, the survey questions assessed one’s ability to acquire financial assistance, whether care was not sought or was delayed due to lack of funds and the use of financial advice/consultation services.

#### Accommodation

The accommodation dimension was characterised as the alignment between health service provision procedures and the population’s logistical needs by focusing on service organisation/processes and experiences from patient-provider interaction (Supplemental Table 7). Timing measures such as waiting times, service hours, appointment times, and time delays for treatment and test results dominated service process metrics. Patient experiences used to assess the accommodation dimension included satisfaction with appointment scheduling procedures, the ease of communication processes e.g. the existence of different communication channels for booking appointments, referral procedures, and the convenience in interacting with the healthcare facilities and medical professionals (Supplemental Table 7). Retrospective/secondary analysis studies characterised the accommodation dimension using an outcome oriented approach and involved healthcare utilisation proxies including antenatal care visits, family planning, immunisation rates and facility deliveries.

#### Acceptability

Congruence between cultural/personal expectations and the appropriateness of the rendered healthcare services was used to define acceptability (Supplemental Table 8). Survey-based studies used questions that directly assessed patient/population experiences with interpersonal aspects and the broader healthcare system structure in the delivery of services. Questions on the interpersonal aspects included assessing whether the services were provided respectably, if privacy/confidentiality was facilitated during the service dispensation and whether the communication was complete and issued appropriately (Supplemental Table 8). For the healthcare system structure, surveys assessed the level of confidence in the services and treatments offered and whether lack thereof, was a significant barrier to seeking formal care and resorting to other care alternatives. In addition, the cultural alignment in the characteristics of the available workforce such as gender was assessed. Retrospective/secondary analysis studies relied on healthcare behavioural metrics to characterise acceptability such as inpatient/outpatient utilisation rates, facility bypassing and inappropriate service use (Supplemental Table 8).

### Composite index

The synthesis of the methodological approaches in the construction of composite indices of access to healthcare resulted in a five-step process; (i) framework adoption to define access dimensions and selection of corresponding indicators, (ii) standardisation of indicators to ensure comparable measurement scales, (iii) defining weights across the dimensions to establish relative importance, (iv) aggregation of dimension indicators to generate a single composite index, and (v) performing composite index robustness and validation checks.

#### Frameworks of access

Three frameworks were adopted to select the indicators and define the multi-dimension index of access, and 9 studies provided this information. These included the Penchansky & Thomas [4] framework (4 studies), one study was based on the Lancet Commission on Global Surgery (LCoGS) framework, and four studies based their definitions on previously conducted qualitative studies focused on barriers to access in their study areas, while 10/19 studies did not explicitly state the framework adopted in their definition of access to healthcare dimensions.

#### Selection of indicators

Based on the selected framework and definition of access dimensions, the first step entailed the selection of the indicators for the dimensions. Let $\:{\{D}_{i}:i=\text{1,2},\text{3,4},5\}$ represent dimension $\:i$. For each $\:{D}_{i}$, the respective studies identified a relevant indicator, or a set of indicators, $\:\{{x}_{j}:j=\text{1,2},3,\dots\:,n\}$ (Supplemental Tables 4–8). A dimension could be represented by either a single indicator or multiple indicators. For example, Rajeshwari [31] used Thiesen polygons to represent accessibility, whereas Debnath et al. [25] used distance, ambulance functionality, and road network connectivity to represent accessibility.

The data used to represent these indicators was derived mainly from two sources, either through primary data collection (survey) or the use of existing data. Survey-based studies used indicators based on either a single question or set of questions per dimension, scored using Likert scales or ranked options. Retrospective and secondary studies relied on secondary data sources such as relevant ministries of health to obtain lists of healthcare facilities and staffing, Health Management and Information Systems (HMIS) for healthcare utilisation metrics, national population census data, and existing national surveys such as the National Family Health Survey (NFHS) to obtain indicators on population characteristics as per their dimension definitions. Consequently, dimension indicators for retrospective studies were in terms of proportions/percentages per unit of analysis.

#### Indicator normalisation and standardisation

In some studies, the scale of measurement varied across indicators/dimensions based on data collection method. Consequently, the studies utilised different approaches to normalise/standardise the indicators to a common scale of measurement predominantly using variations of the min-max normalisation formula. Supplemental Table 9 shows the normalisation approaches used for each study. After normalisation, some of the studies with multiple indicators per dimension constructed a sub-composite indicator for each of the dimensions by averaging the normalised scores [26, 30, 32–34] while others normalised the multiple indicators but did not construct a composite index of the respective dimensions [25, 28, 29, 35]. Likert-scale scores on multiple question indicators were also normalised in survey-based studies. For example, Blanco et al. [28] summed the normalised Likert scale scores across survey questions for each dimension to create composite indices for each dimension. It is these sub-composite indicators that were combined into the access composite index.

#### Weighting

With the normalised indicators/dimension scores, the studies defined the relative importance of each dimension using a weighting scheme. The weighting schemes were heterogeneous ranging from equal weights [26–28, 30–32, 34–40] in 13 studies, Principal Component Analysis (PCA) [29, 33, 41, 42] in 4 studies, Analytical Hierarchy Process (AHP) [43] in only 1 study and based on priority areas of a national programme [25] in 1 study (Supplemental Table 9). The use of equal weighting was justified across studies as an attempt to maintain the simplicity, transparency and interpretability of the results, whereas PCA was utilised as an objective data-based algorithm to allocate weights based on the amount of variance explained by the indicators/dimensions. The studies that utilised AHP and national priority areas to define dimension weights aimed to incorporate the contextual importance of the access dimensions within their study area.

#### Aggregation models

With the dimensions defined and weights derived, four models were used to bring these data and weights together to define the composite index of access. Arithmetic mean (Eq. 1) was the most utilised aggregation method (10 studies), followed by summation (Eq. 2), in six studies. Studies using these two techniques argued their simplicity in both the implementation and interpretation (Supplemental Table 9). However, under limitations, the studies highlighted that both techniques are fully compensatory in nature - a low score in one of the dimensions is offset by a high score in another, which might not be the case in the context of healthcare access; a severe low score in a single access dimension such as affordability can deter access altogether.

1 $$\:\begin{array}{c}{A}_{s}=\frac{1}{n}\lfloor\sum_i^n\:{\omega\:}_{i}\:{D}_{i}\rfloor\:\:\:\:\:\:\:\:or\:\:\:\:\:\:\:\:\:{A}_{s}=\frac{1}{n}\lfloor\sum_i^n\:{\omega\:}_{i}\:{x}_{i}\rfloor\end{array}$$

2 $$\:\begin{array}{c}{A}_{s}=\sum_i^n\:{\omega\:}_{i}\:\:{D}_{i}\:\:\:\:\:\:\:\:or\:\:\:\:\:\:\:\:{A}_{s}=\sum_i^n\:{\omega\:}_{i}\:\:{x}_{i}\end{array}$$

where $\:{A}_{s}$ is the composite index of access for unit $\:s$ (individual/household/polygon), $\:{\omega\:}_{i}$ is the weight for dimension, $\:{D}_{i}$ (or normalised indicator $\:{x}_{i}$), and $\:n$ is the number of dimensions (or normalised indicators). For equal weighting, $\:{\omega\:}_{i}=1$, for all elements.

Two studies used the Adjusted Mazziotta-Pareto Index (AMPI) (Eq. 3).

3 $$\:\begin{array}{c}{A}_{s}={\mu\:}_{ri}-(S{D}_{ri}\times\:C{V}_{ri})\end{array}$$

Where $\:{\mu\:}_{ri}$ is the mean of the normalised dimension indicators, $\:S{D}_{ri}$ is the standard deviation of the normalised dimension values. $\:C{V}_{ri}$ is the coefficient of variation i.e. $\:\frac{S{D}_{ri}}{{\mu\:}_{ri}}$. The term $\:S{D}_{ri}\times\:C{V}_{ri}$ acts as penalisation term for variability (imbalance) in the dimension scores. These studies aimed to account for the full compensatory limitation when constructing a composite index of access [27, 37]. Specifically, the AMPI first calculates the arithmetic mean of the access dimension scores, $\:{\mu\:}_{ri}$, and adds a penalisation term, $\:S{D}_{ri}\times\:C{V}_{ri}$. If the dimension scores are similar, the penalty term tends to zero, whereas if there exists variation (high scores in some dimensions and low scores in others) the penalty term is greater, thus lowering the composite index from the mean.

Similarly, one study aimed to account for the full compensatory limitation using geometric mean (Eq. 4) [26]. The multiplicative nature of the geometric mean allowed for low scores to disproportionately decrease the composite index, furthermore, if any of the dimensions had a score of zero, the access composite index would be equal to zero, reflecting no access to care whatsoever.

4 $$\:\begin{array}{c}{A}_{s}={\left(\prod_i^n{D}_{i}\right)}^{\frac{1}{n}}\:\:\:\:\:\:\:\:\:\:or\:\:\:\:\:\:\:\:\:\:{A}_{s}={\left(\prod_i^n{x}_{i}\right)}^{\frac{1}{n}}\end{array}$$

### Validation

Various validation approaches were undertaken to validate the constructed composite indices; however, validation was not done in about a third of the studies (7, 37%) (Supplemental Table 9). Five studies utilised regression techniques to assess the relationship between the constructed access composite index and a health outcome [25, 36, 39, 41, 42]. For example, Weinstein et al. [42] and Fleetcroft et al. [39] fitted regression models to assess changes in the risk of COVID-19 cases and asthma admissions, respectively, with respect to different levels of the access composite index. A further six studies used measures of association such as Analysis of Variance (ANOVA), t-tests, correlation, and chi-square tests to assess the association between the developed composite access index and demographic characteristics [27, 28, 33, 35, 38]. In one study [26],, a robustness sensitivity was done by utilising different aggregation techniques and different proxies of access dimensions.

## Discussion

Adequate access to quality healthcare services on all access dimensions is critical towards achieving SDG targets [3]. To identify gaps and inequities as a starting point towards improving and monitoring progress of Universal Health Coverage (UHC) requires a multidimensional lens for a more comprehensive view across a population [4–6, 17, 18] based on robust methods. Our systematic literature review identified 19 studies that have been used to create a composite multi-dimensional index of access. Seven in ten (13/19) studies were conducted in India or the USA, and only a quarter of all studies incorporated all the five dimensions of access when creating the composite index. Notably, none of the studies were conducted in sub-Saharan Africa (sSA), despite the region having the greatest need for improved healthcare access. There was substantial heterogeneity in how the indicators used to represent dimensions were defined and measured, and how dimensions were combined. These findings underscore the need for well documented robust approaches for assessing the level of access to healthcare services in a multidimensional manner.

There is a growing attention given to analysis of multi-dimensional composite indices, 68.4% of all the studies identified were conducted after 2020. This signals a concrete shift in focus on the multidimensional approach of assessing the level of access. This resonates with both the calls to identify and contextualise barriers in accessing quality healthcare services as a tool towards the promotion of healthy lives for all [3] and the rapid developments in Geographic Information Systems (GIS), availability of remotely sensed data on various population and healthcare characteristics.

This review highlights that the choice of framework of access introduced heterogeneities in the selection of indicators used to represent dimension across the studies. As a result, there were many instances where the same indicator was used for different dimensions across different studies. For example, Cabrera-Barona et al. [41] used waiting times as a measure of availability whereas waiting times were utilised as a measure of accommodation in other studies [28, 33, 34, 36, 39, 40]. This was also observed for healthcare utilisation metrics such as ANC attendance, facility delivery and immunisation coverage where they were used as indicators for the affordability dimension by Kumari & Raman [29], accommodation dimension by Banu & Biswas [32] and Majumder et al. [30], and acceptability dimension by Weinstein et al. [42]. These inconsistencies justify the need for guidelines to operationally define access dimensions and establish consensus indicator-dimension matching, consequently ensuring cross-study comparability of composite access indices.

While surveys (primary data collection) or secondary data were used across the studies, data derived from survey-based studies provided context-specific data, which was flexible and allowed direct measurement (through tailored questions explicitly designed to capture each domain) at high spatial resolution. However, this granularity in primary data collection has inherent trade-offs; higher spatial resolution significantly increases costs (time, human resource and financial costs), in addition to other limitations in surveys of subjectivity and reporting bias. This might explain why all survey-based studies were in High-Income Countries (HICs) and only three studies were in Low-and-Middle-Income countries (LMICs), specifically India (*n* = 2) and Ecuador (*n* = 1). Notably, despite collecting data at granular level (individual/household), none of the survey-based studies utilised advanced approaches such as model-based geostatistical methods [44] to generate continuous, pixel-level estimates of their composite access indices. Such estimates would allow the granular identification of hotspots of marginalised populations and allow for adoption of targeted interventions and polices towards addressing access inequalities. On the other hand, majority of the studies used secondary data due to their cost-effectiveness, however, the indicators and results were summarised at aerial units (administrative boundaries), masking local heterogeneities. In addition, limitations in using secondary data include biases e.g. from missingness or data aggregation and data availability challenges especially for the accommodation and acceptability dimensions, with the latter resulting in reliance on utilisation proxies.

The multidimensionality of access introduces the need for defining relative importance across the dimensions of access. Across the studies included in this review, the choice of weighting approach was based on simplicity and transparency, data-based approaches and approaches that incorporate the contexts of the study areas. Each of the approaches has its own strengths, limitations, justification/recommendations for use and implications on the results, which are well documented in health literature [45–47]. In the context of access dimensions, the drawback of equal weights is that it ignores contextual variability in barriers to access. For example, accessibility (distance/travel time) might be the more important barrier in rural areas compared to accommodation (long waiting times) in urban areas. Consequently, allocating equal weights in different contexts might not reflect reality of the different barriers to access. Similarly, PCA ignores the epidemiological importance of the different dimensions as it allocates weights solely based on the statistical variance and correlation exhibited within the data [45]. Furthermore, weights derived from PCA are dataset specific; changes in the dimension indicators may result in different allocation of weights across the access dimensions.

In contrast, the use of AHP and national priority areas to define weights allows for the incorporation of contextual importance of the access dimensions when constructing a composite access index. However, unlike data-driven methods that can handle many indicators, the number of indicators to be included in AHP is limited and concordance on the significance of the dimensions across different stakeholders might vary, while the utility of national programmes is limited to a pre-determined timeframe.

Existing literature on the dimensions of access and the subsequent quantification emphasises on the concept of interrelatedness and non-substitutability of access dimensions [4–6, 16]. Consequently, this should be integrated in the choice of an aggregation technique ensuring that the focus is on penalising low scores across the dimensions as opposed to rewarding those with higher scores, which is consistent with the emphasis on efforts to eliminate barriers of access to healthcare services as opposed to facilitators [3]. Studies using compensatory aggregation techniques where a low score in one of the dimensions is offset by a high score in another are not reflective of the non-substitutability of access dimensions; a severe low score in a single access dimension such as affordability can deter access altogether. Furthermore, aggregation techniques such as the AMPI that reward a system where access dimensions are balanced and penalises a system constituting of high and low performance dimensions [48] allow for the prioritisation of resources toward the poor performing dimensions. Similar methods have been utilised in the creation of the UHC index that utilise the geometric mean to aggregate the respective components [49].

Validation of composite indices is an important step in computing useful composite indices [45, 46]. Across studies in this review, validation was done by assessing the association between the constructed index and a given health outcome or by contextualising of the areas with low/high access indices. A robustness check was also undertaken to investigate the volatility of the index to changes in indicators or weighting schemes as done by Mukherji at al. [26]. These techniques are within the general guidelines for constructing composite indices [45, 46]. However, they are not sufficient. The ideal scenario would be to conduct a cross validation of the index against an established benchmark or existing local inventories as done by Membele et al. [50] who developed a high spatial resolution vulnerability index and cross-validated its coverage to the municipal list of vulnerable households. Alternatives include engaging local stakeholders to assess the validity of the distribution of the composite index. For robustness, sensitivity to changes in the construction process such as dimension indicators and weighting/aggregation algorithms and uncertainty measures should be included [45, 46]. It is important to note that the observed methodological challenges persist in other disciplines such as climate vulnerability indices [51, 52] and socioeconomic deprivation scores [53, 54] and are not unique to healthcare access indices. This underscores the need for cross-disciplinary methodological learning.

While isolated assessment of access dimensions allows for granularity to identify and implement dimension-specific interventions that address specific access barriers, composite indices reveal systemic complexities across the dimensions that the siloed approach obscure. Specifically, composite indices capture the co-occurrence of multiple barriers, dimension interrelatedness, and emergent access gaps (deficiencies resulting from the dimension interactions despite no single dimension appearing deficient). This hierarchical approach that identifies lagging areas in access using composite indices and decomposes the composite indices to pinpoint the deficient dimensions allows the composite score to serve both as a monitoring and diagnostic tool. Such tools are particularly essential in sSA (and other LMICs), where vast literature exists on deficiencies in the single access dimensions, but systemic analyses remain rare, to guide policymakers on how these barriers interact to constrain access to healthcare facilities.

### Recommendations

To ensure robust composite indicators that are context-specific and more comprehensive across literature, we recommend future studies to prioritise four key areas. One, explicitly adopting a definition framework such as the Penchansky & Thomas [4] or Levesque et al. [6] or an outcome-specific framework as done by Zadey [27] depending on the use-case. This will aim to ensure consistency in indicator allocation within the dimensions across studies. Two, dimension weights should be unequal to represent the variation of the barriers to access in different contexts and the weighting algorithms should incorporate both data-driven and contextual aspects to enhance robustness. For example, a low-income country facing high out-of-pocket health expenditures might intentionally assign greater weight to affordability over other dimensions, not because affordability is inherently more important, but because addressing financial barriers represents the most pressing policy priority in that country. Three, studies should prioritise non-compensatory aggregation approaches to penalise critical deficiencies in any of the dimensions as this reflects the interrelatedness and non-substitutability of healthcare access dimensions. Finally, multiple validation and robustness checks on the constructed indicator should be carried out including use of benchmarks, local stakeholders and associations with health outcomes.

### Strengths and limitations

This review provides an evidence base on the construction of composite healthcare access indices through a comprehensive synthesis of existing literature adhering to the PRISMA guidelines for transparency (Supplementary Table 10). To our knowledge, this is the first review of the methods used to more comprehensively define access to healthcare incorporating all the dimensions of access. The review is not without limitations. First, the review considered only articles published in English, which may have excluded relevant papers in non-English speaking countries. Two, the exclusion of grey literature such as institutional reports may have missed some methodological insights. Three, the review does not capture the universal methods used to create composite indices as the focus is on access-related composite indices. Lastly, due to the limited sample size of included studies and the primary objective of this review—to synthesize methodological approaches for constructing composite access indices across all healthcare contexts - a formal sub-analysis comparing access conceptualisation between general and specialized services was not feasible. While the core dimensions of access (availability, accessibility, accommodation, affordability, acceptability) are universal, their operationalisation (choice of indicators and relative importance) may differ based on the services sought representing an opportunity for future research.

## Conclusion

The increasing global focus on improving access to quality healthcare services underscores the need for multidimensional and comprehensive approaches for monitoring progress, which is critical to identify lagging areas and target interventions to address any existing disparities in healthcare access. This systematic literature review highlights the potential and limitations of the diverse existing methodologies in literature for measuring access to healthcare facilities in a multidimensional lens. The goal for future studies should be to adopt robust context-specific approaches that utilise clearly defined access frameworks and non-compensatory aggregation methodologies that offer a more accurate and actionable assessment of access to healthcare facilities. Ultimately, equitable healthcare access is dependent on addressing all dimensions of access, as systemic deficiencies in any single dimension can impede overall progress.

## Supplementary Information

Supplementary Material 1.

## Data Availability

The dataset(s) supporting the conclusions of this article is(are) included within the article (and its additional file(s)).

## Abbreviations

AHP: Analytical hierarchy process
AMPI: Adjusted Mazziotta-Pareto index
ANOVA: Analysis of variance
COVID: 19-coronavirus disease 2019
GIS: Geographic information systems
HIV: Human immunodeficiency virus
HMIS: Health management and information systems
NFHS: National family health survey
PCA: Principal component analysis
PRISMA: Preferred reporting items for systematic reviews
SDG: Sustainable development goal
UHC: Universal health coverage

## Declarations

### Patient and public involvement

Patients and/or the public were not involved in the design, or conduct, or reporting, or dissemination plans of this research.
