# Supplementary material for: Methods for modelling composite indices of access to healthcare facilities: a systematic literature review
Source: Popul Health Metr. 2025 Nov 21;23:73. doi: 10.1186/s12963-025-00432-7 (PMC12706922; doi:10.1186/s12963-025-00432-7)
Supplement: Supplementary file 1 — Supplementary Material 1. [file 12963_2025_432_MOESM1_ESM.docx]

**Search strategy**

*EBSCOhost (CINAHL):*

(((composite N3 metri*) OR (composite N3 indicat*) OR (composite N3 score) OR (composite N3 ind*)) AND ((health* N3 access*) OR (health* N3 inequ*)))

Limiter - Language: English

*Google Scholar*

((composite AND metr*) OR (composite AND indicat*) OR (composite AND score) OR (composite AND ind*)) AND ((health* AND access*) OR (health* AND inequ*))

Limiter - Language: English

*Ovid (Embase):*

((composite adj3 metri*) or (composite adj3 indicat*) or (composite adj3 score) or (composite adj3 ind*)) AND ((health* adj3 access*) or (health* adj3 inequ*))

Limiter - Language: English

*Ovid (Medline):*

((composite adj3 metri*) or (composite adj3 indicat*) or (composite adj3 score) or (composite adj3 ind*)) AND ((health* adj3 access*) or (health* adj3 inequ*))

Limiter - Language: English

*PubMed:*

(("composite index"[tiab:~3]) OR ("composite indicator"[tiab:~3]) OR ("composite score"[tiab:~3]) OR ("composite index") OR ("composite indicator") OR ("composite score")) AND (("health* access*") OR ("health* inequ*")) AND (English[Language])

*Web of Science:*

TS=((((composite NEAR/3 metric) OR (composite NEAR/3 ind*) OR (composite NEAR/3 indicator) OR (composite NEAR/3 score*)) AND ((health* NEAR/3 access*) OR (health* NEAR/3 inequ*))))

Limiter - Language: English

*Web of Science (Medline):*

TS=((((composite NEAR/3 metric) OR (composite NEAR/3 ind*) OR (composite NEAR/3 indicator) OR (composite NEAR/3 score*)) AND ((health* NEAR/3 access*) OR (health* NEAR/3 inequ*))))

Limiter - Language: English

**Supplemental Table 1**: A summary on the data variables extracted for included studies

| **Bibliography Details** | Author (full) – Paper reference |
| --- | --- |
|  | Author – First name of author |
|  | Type of publication |
|  | PubMed ID (PMID) |
|  | PubMed Central ID (PMCID) |
|  | DOI number |
|  | Databases |
|  | Region/Country |
| **Hospital care facets included (and datasets used)** | Availability (datasets used) |
|  | Affordability (datasets used) |
|  | Acceptability (datasets used) |
|  | Accommodation (datasets used) |
|  | Geographic accessibility (datasets used) |
| **Model used and validation** | Model in abstract |
|  | Model in full text |
|  | Model category |
|  | Code/software availability (Y/N) |
|  | Model validation method |
|  | Spatial/temporal resolution |
| **Additional datasets/covariates** | Any additional datasets e.g. tuning parameters |
| **Limitations and recommendations** | Limitations |
|  | Recommendations |

**Supplemental Table 2**: Quality Appraisal Tool used to assess the quality of the included studies

|  | **Criterion** | **Considerations** | **Score considerations (0, poor to 2, good)** |  |
| --- | --- | --- | --- | --- |
| 1. **Screening questions** | | | | |
| 1 | Are the aims and objectives clear? | Are the research questions and modelling objectives clearly defined? | 0 Not stated  1 Stated but vague  2 Stated and focussed | Screening: max 6 points |
| 2 | Is the setting and population clearly defined? | Does the paper clearly state the setting (e.g. geographical location, hospital definition)? | 0 Not stated  1 Stated but vague or details missing  2 Stated and focussed |  |
| 3 | Are the outcome measures defined and answer the research question? | Does the paper clearly define the outcomes of interest?  Do the outcomes correspond to the research question? | 0 Not stated, very unclear or not suited to research question  1 Stated but details missing or not directly aligned with research question  2 Stated, all necessary details stated, and aligned with research question |  |
| 1. **Assessing the validity of the model** | | | | |
| 4 | Are the model structure and time horizon clearly described and appropriate for the research question? | Is the model structure clearly reported and appropriate for the research question? | 0 Not appropriate model structure, or poor/no description of model  1 Incomplete description, and/or appropriate in part for research question  2 Complete and reproducible, appropriate structure and time horizon | Methods: max 14 points |
| 5 | Are the modelling methods appropriate for the research question and adequately described? | Were the modelling methods clearly described, and suited to the research question? | 0 Not appropriate model structure, or poor/no description of methods  1 Incomplete description, and/or appropriate in part for research question  2 Complete and reproducible, appropriate method |  |
| 6 | Are the parameters, ranges and data sources specified? | Are all parameters and their ranges reported?  Are the data sources for parameters reported? | 0 Poorly reported  1 Some information missing  2 Complete reporting of parameters, ranges and data sources |  |
| 7 | Are any assumptions explicit and justified? | Are all assumptions explicit and justified? | 0 Not reported  1 Explicit  2 Explicit and justified |  |
| 8 | Is the quality of data considered? | Are data limitations discussed? Are any of the sources known to the reviewer to be inappropriate (e.g. do not match the parameter, are outdated, or known to be poor quality)? | 0 No sources or uncertainty  1 Partially addressed, and/or some data inappropriate  2 Fully addressed |  |
| 9 | Is the method of fitting described and suitable? | Is the method of fitting/calibrating the model clearly described?  Is the method of model fitting/calibration suitable? | 0 Not done, unsuitable method or poor/no description  1 Incomplete description or method not optimal  2 Complete description and suitable methods |  |
| 10 | Has the model been validated? | Has an assessment of validity of the results been made by comparing across one or more different model structures, or against a validation data set? | 0 Not considered  1 States criteria for validation  2 Validation undertaken |  |
| 1. **Assessing results and study conclusions** | | | | |
| 11 | Have the results been clearly and completely presented, with a range of uncertainty? | Have the outcome values and their uncertainty ranges been reported?  Do the results match the objectives? | 0 Not reported, very unclear or not suited to research question  1 Stated, but ranges or planned sensitivity analyses missing and/or not directly aligned with research question  2 Values and ranges and planned sensitivity analyses reported and aligned with research question. | Results and Conflicts: max 6 points |
| 12 | Are the results appropriately interpreted and discussed in context? | Are the results of the study discussed in context and is generalisability considered?  Are possible biases and limitations discussed? | 0 No/poor discussion  1 Some discussion but key points, limitations or context missed  2 Full discussions of key points in context, generalisability considered, limitations discussed |  |
| 13 | Are the funding source and conflicts of interest reported? | Is the funding and the role of the funder clearly stated?  Is there a conflict-of-interest statement? | 0 No statement of funding or conflicts  1 Funding or conflicts reported  2 Funding and conflict statement |  |

**Supplemental Table 3**: Article appraisal scores awarded for each of the 19 included studies

| Author | Aims and Objectives | Setting and population | Outcome measure | Model structure | Model Methods Appropriate | Data sources and parameters | Assumptions | Data quality | Model calibration | Model validation | Results completeness | Results interpretation | Funding source | Total |
| --- | --- | --- | --- | --- | --- | --- | --- | --- | --- | --- | --- | --- | --- | --- |
| Al Asfoor (2020 | 2 | 2 | 2 | 1 | 2 | 2 | 2 | 2 | 1 | 1 | 1 | 2 | 0 | 20 |
| Banu & Biswas (2022) | 2 | 2 | 2 | 1 | 1 | 1 | 1 | 1 | 1 | 0 | 1 | 2 | 2 | 17 |
| Bhattacharya & Ghos (2024) | 2 | 2 | 2 | 2 | 2 | 2 | 0 | 2 | 2 | 1 | 2 | 2 | 2 | 23 |
| Blanco et al (2024) | 2 | 1 | 2 | 2 | 2 | 2 | 1 | 2 | 2 | 1 | 2 | 2 | 2 | 23 |
| brown et al (2021) | 2 | 1 | 2 | 1 | 1 | 1 | 0 | 1 | 2 | 0 | 1 | 2 | 2 | 16 |
| Bruzzi et al (2022) | 2 | 1 | 2 | 2 | 2 | 2 | 2 | 1 | 2 | 0 | 2 | 2 | 2 | 22 |
| Cabrera-Barona et al (2017) | 2 | 2 | 2 | 2 | 2 | 1 | 0 | 1 | 1 | 1 | 2 | 2 | 2 | 20 |
| Chatterjee et al (2022) | 2 | 2 | 2 | 2 | 2 | 2 | 2 | 1 | 1 | 2 | 2 | 2 | 2 | 24 |
| Cunningham et al (1998) | 2 | 1 | 1 | 1 | 1 | 1 | 1 | 1 | 1 | 1 | 2 | 2 | 0 | 15 |
| Debnath et al (2023) | 2 | 2 | 2 | 1 | 2 | 1 | 0 | 0 | 1 | 2 | 2 | 2 | 2 | 19 |
| Dulin et al (2010) | 2 | 2 | 2 | 2 | 2 | 1 | 0 | 1 | 2 | 0 | 2 | 2 | 2 | 20 |
| Fleetcroft et al (2016) | 2 | 2 | 2 | 1 | 1 | 2 | 1 | 1 | 0 | 2 | 2 | 2 | 2 | 20 |
| Kumari & Raman (2022) | 2 | 2 | 2 | 2 | 2 | 2 | 1 | 1 | 2 | 0 | 2 | 2 | 1 | 21 |
| Majumder et al (2023) | 2 | 2 | 2 | 2 | 2 | 1 | 1 | 1 | 2 | 0 | 2 | 2 | 1 | 20 |
| Mukherji et al (2024) | 2 | 2 | 2 | 1 | 1 | 2 | 0 | 1 | 0 | 1 | 2 | 2 | 1 | 17 |
| Rajeshwari et al (2024) | 2 | 1 | 1 | 1 | 1 | 1 | 0 | 1 | 0 | 0 | 2 | 2 | 0 | 12 |
| Verma et al (2012) | 2 | 2 | 1 | 1 | 1 | 1 | 1 | 1 | 1 | 1 | 2 | 2 | 2 | 18 |
| Weinstein et al (2021) | 2 | 2 | 2 | 2 | 2 | 2 | 0 | 1 | 1 | 2 | 1 | 1 | 2 | 20 |
| Zadey (2021) | 2 | 2 | 2 | 2 | 2 | 2 | 2 | 2 | 1 | 2 | 2 | 2 | 2 | 25 |

**Supplemental Table 4**: Accessibility/Geographic access: the definition and data used across the included studies that incorporated the geographic access dimension (n = 9)

| **Author** | **Definition** | **Dataset** |
| --- | --- | --- |
| 1. Al Asfoor (2020) | Accessibility refers to the relationship between the location of providers of healthcare and the location of clients or receivers. | - How long does it usually take you to get to the health centre? - In the past 12 months, did you cancel or delay a visit to the health centre because you had no transport? - In the past 12 months, did you cancel or delay a visit to the health centre because you could not afford the cost of transport? |
| 1. Banu & Biswas (2022) | Penchansky & Thomas (1981) framework* | - % of Villages with Sub-Health Centre within 3 km. - % of Villages with PHC within 10 km |
| 1. Bhattacharya & Ghosh (2024) | Penchansky & Thomas (1981) framework* | - How far is the travel to the healthcare centers? - Are you satisfied with the typical travel time to your healthcare centers? - How difficult is it for you to commute to the healthcare centers? |
| 1. Blanco et al (2024) | Ability to travel to the provider of the health good/service | Survey questionnaire - Likert scale  *Statements about the geographic dimension*  *1. The service was nearby*  *2. It took a long time to get there*  *3. Going and coming back was easy* |
| 1. Cabrera-Barona et al (2017) | The distance to the service´s location | Survey questionnaire - Perceived travel time |
| 1. Debnath et al (2023) | Penchansky & Thomas (1981) framework | - Distance to PHC from SHC, - PHC connected with Pucca Road, - Ambulance functionability, - Facility accessible throughout the year by transport |
| 1. Rajeshwari (2024) | Area served per health care facility | Thiesen polygon - 1km |
| 1. Verma et al (2012) | Distance from home | Survey questionnaire |
| 1. Zadey (2021) | the Lancet Commission on Global Surgery (LCoGS) | Proportion (%) of the rural population within 120 minutes (2 hours) of their nearest surgical care facility |

**Supplemental Table 5**: Availability: the definition and data used across the included studies that incorporated the availability access dimension (n = 14)

| **Author** | **Definition** | **Dataset** |
| --- | --- | --- |
| 1. Al Asfoor (2020) | Availability of services and medicine (Discussing medications, usual source of care, availability of blood tests and appointments with GPs, specialists and other health professionals). | - In the past 12 months, has a doctor talked to you about medications you take, including the ones prescribed by your doctor? - Do you have a health centre or a personal GP or doctor that you consult? - During the last year, were you prescribed a medicine that you did not get? - If the doctor asks you to get a blood or other test, is it always available at the health centre? - If you need to see a dietitian, physiotherapist, or another health professional, are they always available? - During the last year, have you had difficulty getting medical treatment because there was no doctor or another health professional available? |
| 1. Banu & Biswas (2022) | Penchansky & Thomas (1981) framework* | No. of Health centers and staffing per 100,000 |
| 1. Blanco et al (2024) | - Institutional availability: the existence of institutions providing health services from which individuals with health needs can choose. - Material availability: the presence of adequate resources (equipment, staff) | Survey questionnaire - Likert scale  *Statements about the material availability dimension*  *1. The necessary implements were available*  *2. The professional was qualified to address the inquiry* |
| 1. Bruzzi et al (2022) | number of residential beds | No. residential social-health residential beds for the elderly and people with disabilities (for inhabitant) |
| 1. Cabrera-Barona et al (2017) | The appointment waiting time (in hours) to receive healthcare | Survey questionnaire - Waiting times |
| 1. Chatterjee & Sarkar (2022) | Distribution of health facilities | Spatial distribution Health care facilities, number of beds |
| 1. Cunningham et al (1998) | Based on a previous qualitative study | Availability of Emergency care, Hospital care, Urgent care |
| 1. Debnath et al (2023) | Penchansky & Thomas (1981) framework | - PHC functioning 24x7 - Number of Beds per PHC - PHC provided ANC care or not - Ratio of ANM and PHC, Ratio of Medical Officer and PHC - Operation theatre available at PHC, Normal delivery services availability - Power Availability & Running Water Availability in the PHC |
| 1. Kumari & Raman (2022). | Based on a review of previous studies in the study area | No. of hospitals, doctors and beds per 100k of population |
| 1. Majumder et al (2023) | Penchansky & Thomas (1981) framework* | - Hospitals, Doctors and beds per 100,000, - No. of family welfare centers and PHC serving the district |
| 1. Mukherji et al (2024) | Service capacity | - % of functioning facilities available in a district providing round-the-clock health care as per norms of Indian Public Health Standards, - Beds available in a district as a % of the norm specified by the Indian Public Health Standards, - % of essential medicines (out of the list of the Indian Public Health Standards) available in primary health-care centres |
| 1. Rajeshwari (2024) | Ratio of health care facility with the spatial distribution of the population | Ratio of population to various health care facilities |
| 1. Verma et al (2012) | Availability of services - Doctors, medicine, EC services, treatment | Survey questionnaire |
| 1. Zadey (2021) | Surgical Capacity – met surgical need | The ratio of observed surgical rates to the threshold of 5000 surgeries per 100,000 people |

**Supplemental Table 6**: Affordability: the definition and data used across the included studies that incorporated the affordability access dimension (n = 16)

| **Author** | **Definition** | **Dataset** |
| --- | --- | --- |
| 1. Al Asfoor (2020) | Affordability of consultation and medicines | - In the past 12 months, did you postpone or abstain from a visit because you could not afford it? - In the past 12 months, was there a time you did not take a medication because you could not afford it? |
| 1. Banu & Biswas (2022). | Penchansky & Thomas (1981) framework* | - Households covered by any scheme of health expenditure support - Type of ward; Paying general, Paying special, - Per capita household’s expenditure during last 365 days |
| 1. Bhattacharya & Ghosh (2024) | Penchansky & Thomas (1981) framework* | - Was there a time in the past 12 months when you had to borrow money or take a loan to visit a doctor? - Was there a time in the past 12 months when you needed to see a doctor but could not because of cost? |
| 1. Blanco et al (2024) | Ability to afford or finance health care, conditioned by their personal and family income, their affiliation or not to health insurance, the extent of outlays they must make to get health care as well as the potential need to seek loans from formal institutions like banks or informal sources such as relatives and friends | Survey questionnaire - Likert scale  *Statements about the economic dimension*  *1. You could not afford or continue the treatment because it was expensive*  *2. The transportation cost was low* |
| 1. Brown et al (2021) | Based on a previous qualitative study | - Dealing with insurance companies, - Out-of-pocket costs, - No health insurance, - Coverage issues with insurance, - Getting medical assistance, - Type of insurance accepted by breast cancer specialist, - Financial hardship, - Financial advice or consultation - Availability of patient services |
| 1. Bruzzi et al (2022) | Household spending | Family health expenditure (Eur) |
| 1. Chatterjee & Sarkar (2022) | Standard of living | - Household Healthcare Utilization & Expenditure in India - Material Living Condition Index - Economic and Physical Safety Index |
| 1. Cunningham et al (1998) | Based on a previous qualitative study | Covering the cost of care no problem,  Not going without care because of expense |
| 1. Debnath et al (2023) | Penchansky & Thomas (1981) framework* | Median income as a proxy of the socio-economic level of municipalities |
| 1. Dulin et al (2010) | Socioeconomic status and Insurance status | Median household income, Uninsured or Medicaid |
| 1. Kumari & Raman (2022). | Based on a review of previous studies in the study area | - households covered by a health scheme/insurance, - mother’s full antenatal care, - institutional birth delivery, - children aged 12–23 months fully immunized, - Average expenditure on non-communicable diseases |
| 1. Majumder et al (2023) | Penchansky & Thomas (1981) framework* | - % of households where at least one member has health insurance, - % of mothers who had full antenatal care, - Average out of pocket expenditure per delivery in public health facility - Per capita household's expenditure during last 365 days |
| 1. Mukherji et al (2024) | Financial risk protection | - % of the population in a district covered by a health insurance scheme |
| 1. Verma et al (2012) | Treatment cost affordable | Survey questionnaire |
| 1. Weinstein et al (2021) | Insurance coverage rate | (%) people with health insurance |
| 1. Zadey (2021) | the Lancet Commission on Global Surgery (LCoGS) | Proportion (%) of households with at least one surgical hospitalization in the last 365 days facing catastrophic health expenditure (CHE) out of all such surgery-seeking households |

**Supplemental Table 7**: Accommodation: the definition and data used across the included studies that incorporated the accommodation access dimension (n = 10)

| **Author** | **Definition** | **Dataset** |
| --- | --- | --- |
| 1. Al Asfoor (2020) | Organisational access (Phone accessibility, appointment availability, clinic waiting time, appointment waiting time) | - When I call my GP, the time it takes to talk to someone? - I know how to get evening, night and weekend appointments? - Did you need to make an appointment for this visit? If you needed to make an appointment for this visit, how long did you wait for this appointment? - How long did you wait between the time you arrived and the time you were seen by a GP? - Were you referred to a specialist during the last 12 months? If yes, how long did you have to wait for the appointment with the specialist? |
| 1. Banu & Biswas (2022). | Penchansky & Thomas (1981) framework* | - Users who received follow-up services for sterilization and IUD within 48 h, - Pregnant women who had antenatal check-up in first trimester, - PHCs functioning on 24 X 7 h basis |
| 1. Bhattacharya & Ghosh (2024) | Penchansky & Thomas (1981) framework* | - How satisfied are you with how easy it is to get in touch with your doctor(s)? - How satisfied are you with how long you have to wait to get an appointment? - How satisfied are you with how convenient your doctor’s clinic hours are? - How satisfied are you with the commute to the healthcare centers? |
| 1. Blanco et al (2024) | Ability to overcome the bureaucratic requirements necessary to receive health care, such as meeting the opening hours of health centers, facing waiting times, dealing with the method of scheduling appointments | Survey questionnaire - Likert scale  *Statements about the geographic dimension*  *1. Communication by phone (WhatsApp or call) or email was possible*  *2. Scheduling appointments was easy*  *3. There was an extensive period for the appointment day*  *4. Administrative procedures were complicated*  *5. The waiting room time was excessive*  *6. The service hours were suitable*  *7. The administrative staff was friendly* |
| 1. Brown et al (2021) | Based on a previous qualitative study | Availability of treatment needed, Clinic hours, Unsure about which doctor to see, Waiting for appointments, Waiting for test results, Waiting for treatment or surgery, Referrals to breast cancer specialists, Scheduling appointments with breast cancer specialists |
| 1. Cunningham et al (1998) | Based on a previous qualitative study | Convenience: Office hours convenient, Location convenient, Contacting provider to ask questions convenient, Accessing specialists convenient |
| 1. Fleetcroft et al (2016) | Sizmur S. Composite domain markers for GPS. Oxford: Picker Institute Europe;2012. | In the past 6 months, how easy have you found getting through on the phone?  Think about the last time you tried to see a doctor fairly quickly. Were you able to see a doctor on the same day or in the next 2 weekdays the GP surgery or health centre was open?  Last time you tried to, were you able to get an appointment with a doctor more than 2 full weekdays in advance? |
| 1. Majumder et al (2023) | the usefulness and accessibility of various vital healthcare services | - % of children aged 12–23 months fully immunized (BCG,measles, and 3 doses each of the polio and diphtheria vaccine) - % of home deliveries supervised by skilled health personnel (out of total deliveries) - % of births assisted by a doctor/nurse/lady health visitor/auxiliary nurse midwife/other health personnel, |
| 1. Verma et al (2012) | Clinic timings, waiting time, appointment for treatment | Survey questionnaire |
| 1. Weinstein et al (2021) | Healthcare needs met when needed | (%) people who could not use healthcare when needed last year |

**Supplemental Table 8**: Acceptability: the definition and data used across the included studies that incorporated the acceptability access dimension (n = 11)

| **Author** | **Definition** | **Dataset** |
| --- | --- | --- |
| 1. Al Asfoor (2020) | Language, information about illness, privacy, respect | - Has a doctor talked to you in a language you understand? - In the past, did you have difficulty understanding your doctor or healthcare professional? - Have you received written or other information about your illness? - When you are discussing your health conditions or concerns, do you always have privacy? - In the past 12 months, have you ever been treated with disrespect during your visit to the health centre? |
| 1. Banu & Biswas (2022). | Penchansky & Thomas (1981) framework* | - % of villages having Accredited Social Health Activists (ASHA), - Sub-Health Centre with Auxiliary Nursing Midwife (ANM) - PHCs having Lady Medical Office |
| 1. Bhattacharya & Ghosh (2024) | Penchansky & Thomas (1981) framework* | - Have you ever been molested by your healthcare provider? - Do the doctors/hospital staff respect you? - Have you ever been mocked by your healthcare provider? - How satisfied are you with the neighborhoods the doctor’s clinic is in? - How satisfied are you with your doctors? |
| 1. Blanco et al (2024) | The degree of acceptance or confidence an individual has in the healthcare system as a whole | Survey questionnaire - Likert scale  *Statements about the Acceptability dimension*  *1. Considered it wasn’t such a serious issue*  *2. Managed to get medication*  *3. Lacks trust in the healthcare system*  *4. Resorted to alternative medicine* |
| 1. Brown et al (2021) | Based on a previous qualitative study | - Holistic approach to treatment, - Uncertainties of managing treatment relative to other responsibilities, - Learning about alternative forms of treatment, - Using alternative forms of treatment |
| 1. Cabrera-Barona et al (2017) | The main reason the patient chose a healthcare service | Reasons  Confidence in the physician/healthcare service  Recommendation from another person  Has health insurance for that service the service was located close to home |
| 1. Chatterjee & Sarkar (2022) | % of People going outside the block to receive services (If the health care facilities of a block are not sufficient or good enough, people are compelled to travel outside the block for better health care service) | Treated patients' data (IPD/OPD) |
| 1. Dulin et al (2010) | Patterns of emergency department utilisation and primary care safety-net utilisation | Inappropriate emergency department utilisation and use of a primary care safety-net |
| 1. Fleetcroft et al (2016) | Sizmur S. Composite domain markers for GPS. Oxford: Picker Institute Europe;2012. | In the reception area, can other patients overhear what you say to the receptionist?  How helpful do you find the receptionists at your GP surgery or health centre |
| 1. Verma et al (2012) | Doctor and staff behaviour | Survey questionnaire |
| 1. Weinstein et al (2021) | Healthcare utility rate | (%) of people who used health care last year |

**Supplemental Table 9**: Aggregation methods used to create composite index of access across the included studies (n=19)

| **Author** | **Normalisation (and Dimension composite score)** | **Weighting method** | **Method for Composite access index** | **Validation** |
| --- | --- | --- | --- | --- |
| 1. Al Asfoor (2020) | - | Equal weights | Sum of all responses | Regression (Quality of life, glycated haemoglobin) |
| 1. Banu & Biswas (2022) | Normalisation was done as follows for each indicator:  $Ind{ex}_{i}=\frac{X_{d}-X_{min}}{X_{max}-X_{min}}$  The indicators were averaged per dimension to create a single score for each dimension | Equal weights | Arithmetic means  $A_{i}=\frac{\sum D_{i}}{5}$ | None |
| 1. Bhattacharya & Ghos (2024) | Normalisation:  $Ind{ex}_{i}=\frac{X_{d}-X_{min}}{X_{max}-X_{min}}$  The indicators were averaged per dimension to create a single score for each dimension | Principal Component Analysis | Weighted sum  $A_{s}=\sum_{i}^{n} \omega_{i} D_{i}$ | ANOVA (association with demographics) |
| 1. Blanco et al (2024) | Multiple questions per dimension for different health services:  $D_{ius}=\frac{\sum Likert scores}{Max(Likert score)\times no. of questions}$  $D_{ius}$ is the score for dimension i for household u for service s.  Overall scores for each service were the product of the dimension scores of the service  $S_{iu}=\prod D_{ius}$ | Equal weights | Arithmetic mean  $A_{u}=\frac{\sum S_{iu}}{No. of services}$ | ANOVA, Kruskal-Wallis test (association with demographics) |
| 1. Brown et al (2021) | No normalisation  Dimension composite: Arithmetic mean of Likert scale values of questions per dimension | Equal weights | Arithmetic means of Likert scale values | None |
| 1. Bruzzi et al (2022) | The indicator for each dimension was normalised:  $r_{ij}=\left( \frac{x_{ij} - x_{{2.5}^{th}}}{\max- min} \right)60+70$  $r_{ij}$ is the normalised index for dimension j for the ith region, $U_{j}$ and $L_{j}$ are the upper and lower limits of dimension j respectively. The study has single indicators for each dimension | Equal weights | Adjusted Mazziotta-Pareto Index (AMPI)  $I_{i}= \mu_{ri}-(SD_{ri} \times CV_{ri})$  $\mu_{ri}$ is the mean of the normalised dimension indicators, $SD_{ri}$ is the standard deviation of the normalised dimension values for region i  $CV_{ri}$ is the coefficient of variation i.e. ratio of standard deviation to the mean of the normalized dimension values. | None |
| 1. Cabrera-Barona et al (2017) | Calculated accessibility using gravity model  Single indicator for availability and acceptability | Principal Component Analysis | Weighted sum  $A_{s}=\sum_{i}^{n} \omega_{i} D_{i}$ | Regression (Demographics) |
| 1. Chatterjee et al (2022) | Normalisation was done as follows for each indicator:  $x_{i}=\frac{x_{d}-x_{min}}{x_{max}-x_{min}}$ | Equal weights | Arithmetic and Geometric mean | t-test (comparison with survey data) |
| 1. Cunningham et al (1998) | - | Equal weights | Sum of Likert scale responses | Differences (Quality of life) |
| 1. Debnath et al (2023) | Normalisation was done as follows for each indicator:  $x_{i}=\frac{x_{max}-x_{d}}{x_{max}-x_{min}}$ | Weights based on priority areas of a national programme | Weighted arithmetic mean  $A_{i}=\frac{\sum\omega_{i} x_{i}}{\sum\omega_{i}}$ | Regression (Reproductive health outcomes) |
| 1. Dulin et al (2010) | Normalisation (method not given) | Analytical Hierarchy Process | Weighted sum | None |
| 1. Fleetcroft et al (2016) | - | Equal weights | Arithmetic mean | Regression (Admission numbers for asthma) |
| 1. Kumari & Raman (2022) | Normalisation was done as follows for each indicator:  $x_{i}=\frac{x_{d}-x_{min}}{x_{max}-x_{min}}$ | Principal Component Analysis | Weighted arithmetic mean  $A_{i}=\frac{\sum\omega_{i} x_{i}}{n}$ | None |
| 1. Majumder et al (2023) | Normalisation was done as follows for each indicator:  $x_{i}=\frac{x_{d}-x_{min}}{x_{max}-x_{min}}$  Weighted sum for indicators for each dimensions – Principal Component Analysis was used for weighting  $D_{i}=\sum\omega_{i} x_{i}$ | Equal weights | Arithmetic mean  $A_{i}=\frac{\sum D_{i}}{n}$ | None |
| 1. Mukherji et al (2024) | No normalisation  Geometric mean per dimension - equal weighting  $\left( \prod_{i}^{n} x_{i} \right)^{\frac{1}{n}}$ | Equal weights | Geometric mean of all dimensions  $\left( \prod_{i}^{5} D_{i} \right)^{\frac{1}{5}}$ | Sensitivity analysis (arithmetic mean, changing indicators) |
| 1. Rajeshwari et al (2024) | Normalisation (method not specified) | Equal weights | Weighted arithmetic mean | None |
| 1. Verma et al (2012) | - | Equal weights | Weighted arithmetic mean | t-tests (Association with utilisation, demographics) |
| 1. Weinstein et al (2021) | - | Principal Component Analysis | Weighted sum  $A_{s}=\sum_{i}^{n} \omega_{i} D_{i}$ | Regression (Risk of COVID 19) |
| 1. Zadey (2021) | The indicator for each dimension was normalised:  $r_{ij}=\left( \frac{x_{ij} - x_{{2.5}^{th}}}{{x_{{97.5}^{th}} - x}_{{2.5}^{th}}} \right)\times\left( U_{j}-L_{j} \right)$  $r_{ij}$ is the normalised index for dimension j for the ith district, $U_{j}$ and $L_{j}$ are the upper and lower limits of dimension j respectively. The study has single indicators for each dimension | Equal weights | Adjusted Mazziotta-Pareto Index (AMPI)  $I_{i}= \mu_{ri}-(SD_{ri} \times CV_{ri})$  $\mu_{ri}$ is the mean of the normalised dimension indicators, $SD_{ri}$ is the standard deviation of the normalised dimension values for district i  $CV_{ri}$ is the coefficient of variation i.e. ratio of standard deviation to the mean of the normalized dimension values. | Correlation (Surgical burden) |

**Differences between registered protocol and review**

During the screening phase, we made one amendment to the pre-registered protocol. The protocol initially proposed the exclusion of studies focused on surgical and maternal care services, categorizing these as "specialised populations." However, upon detailed review and consensus among the reviewers, we determined that the fundamental dimensions of access (availability, accessibility, accommodation, affordability, and acceptability) as defined by the Penchansky and Thomas (1981) framework are universally applicable, regardless of the clinical service type. The methodological approaches for constructing a composite index in these studies were congruent with the core objective of our review. Therefore, to ensure a comprehensive synthesis of methodological approaches and to avoid an unnecessarily narrow scope, we removed this exclusion criterion.

**Supplemental Table 10:** PRISMA checklist describing where each item has been addressed in the review

| **Section and Topic** | **Item #** | **Checklist item** | **Location where item is reported** |
| --- | --- | --- | --- |
| **TITLE** | | |  |
| Title | 1 | Identify the report as a systematic review. | Page 1 |
| **ABSTRACT** | | |  |
| Abstract | 2 | See the PRISMA 2020 for Abstracts checklist. | Page 1-2 |
| **INTRODUCTION** | | |  |
| Rationale | 3 | Describe the rationale for the review in the context of existing knowledge. | Page 4-5 |
| Objectives | 4 | Provide an explicit statement of the objective(s) or question(s) the review addresses. | Page 5 |
| **METHODS** | | |  |
| Eligibility criteria | 5 | Specify the inclusion and exclusion criteria for the review and how studies were grouped for the syntheses. | Page 6-7 |
| Information sources | 6 | Specify all databases, registers, websites, organisations, reference lists and other sources searched or consulted to identify studies. Specify the date when each source was last searched or consulted. | Page 5 |
| Search strategy | 7 | Present the full search strategies for all databases, registers and websites, including any filters and limits used. | Supplementary file 1 – page 1 |
| Selection process | 8 | Specify the methods used to decide whether a study met the inclusion criteria of the review, including how many reviewers screened each record and each report retrieved, whether they worked independently, and if applicable, details of automation tools used in the process. | Page 6-7 |
| Data collection process | 9 | Specify the methods used to collect data from reports, including how many reviewers collected data from each report, whether they worked independently, any processes for obtaining or confirming data from study investigators, and if applicable, details of automation tools used in the process. | Page 6-7 |
| Data items | 10a | List and define all outcomes for which data were sought. Specify whether all results that were compatible with each outcome domain in each study were sought (e.g. for all measures, time points, analyses), and if not, the methods used to decide which results to collect. | Page 7,  Supplementary file page 2 |
|  | 10b | List and define all other variables for which data were sought (e.g. participant and intervention characteristics, funding sources). Describe any assumptions made about any missing or unclear information. | Supplementary file page 2 |
| Study risk of bias assessment | 11 | Specify the methods used to assess risk of bias in the included studies, including details of the tool(s) used, how many reviewers assessed each study and whether they worked independently, and if applicable, details of automation tools used in the process. | Page 7,  Supplementary file page 3-4 |
| Effect measures | 12 | Specify for each outcome the effect measure(s) (e.g. risk ratio, mean difference) used in the synthesis or presentation of results. | Not applicable |
| Synthesis methods | 13a | Describe the processes used to decide which studies were eligible for each synthesis (e.g. tabulating the study intervention characteristics and comparing against the planned groups for each synthesis (item #5)). | Page 4, |
|  | 13b | Describe any methods required to prepare the data for presentation or synthesis, such as handling of missing summary statistics, or data conversions. | Not applicable |
|  | 13c | Describe any methods used to tabulate or visually display results of individual studies and syntheses. | Page 7 |
|  | 13d | Describe any methods used to synthesize results and provide a rationale for the choice(s). If meta-analysis was performed, describe the model(s), method(s) to identify the presence and extent of statistical heterogeneity, and software package(s) used. | Page 7 |
|  | 13e | Describe any methods used to explore possible causes of heterogeneity among study results (e.g. subgroup analysis, meta-regression). | Not applicable |
|  | 13f | Describe any sensitivity analyses conducted to assess robustness of the synthesized results. | Not applicable |
| Reporting bias assessment | 14 | Describe any methods used to assess risk of bias due to missing results in a synthesis (arising from reporting biases). | Not applicable |
| Certainty assessment | 15 | Describe any methods used to assess certainty (or confidence) in the body of evidence for an outcome. | Not applicable |
| **RESULTS** | | |  |
| Study selection | 16a | Describe the results of the search and selection process, from the number of records identified in the search to the number of studies included in the review, ideally using a flow diagram. | Page 7-8 |
|  | 16b | Cite studies that might appear to meet the inclusion criteria, but which were excluded, and explain why they were excluded. | Not applicable |
| Study characteristics | 17 | Cite each included study and present its characteristics. | Page 9-10 |
| Risk of bias in studies | 18 | Present assessments of risk of bias for each included study. | Supplementary file page 5 |
| Results of individual studies | 19 | For all outcomes, present, for each study: (a) summary statistics for each group (where appropriate) and (b) an effect estimate and its precision (e.g. confidence/credible interval), ideally using structured tables or plots. | Page 10-11 |
| Results of syntheses | 20a | For each synthesis, briefly summarise the characteristics and risk of bias among contributing studies. | Page 11-18 |
|  | 20b | Present results of all statistical syntheses conducted. If meta-analysis was done, present for each the summary estimate and its precision (e.g. confidence/credible interval) and measures of statistical heterogeneity. If comparing groups, describe the direction of the effect. | Not applicable |
|  | 20c | Present results of all investigations of possible causes of heterogeneity among study results. | Not applicable |
|  | 20d | Present results of all sensitivity analyses conducted to assess the robustness of the synthesized results. | Not applicable |
| Reporting biases | 21 | Present assessments of risk of bias due to missing results (arising from reporting biases) for each synthesis assessed. | Not applicable |
| Certainty of evidence | 22 | Present assessments of certainty (or confidence) in the body of evidence for each outcome assessed. | Not applicable |
| **DISCUSSION** | | |  |
| Discussion | 23a | Provide a general interpretation of the results in the context of other evidence. | Page 18-23 |
|  | 23b | Discuss any limitations of the evidence included in the review. | Page 23 |
|  | 23c | Discuss any limitations of the review processes used. | Page 23 |
|  | 23d | Discuss implications of the results for practice, policy, and future research. | Page 24 |
| **OTHER INFORMATION** | | |  |
| Registration and protocol | 24a | Provide registration information for the review, including register name and registration number, or state that the review was not registered. | Page 5 |
|  | 24b | Indicate where the review protocol can be accessed, or state that a protocol was not prepared. | Page 5 |
|  | 24c | Describe and explain any amendments to information provided at registration or in the protocol. | Page 18 |
| Support | 25 | Describe sources of financial or non-financial support for the review, and the role of the funders or sponsors in the review. | Page 25 |
| Competing interests | 26 | Declare any competing interests of review authors. | Page 25 |
| Availability of data, code and other materials | 27 | Report which of the following are publicly available and where they can be found: template data collection forms; data extracted from included studies; data used for all analyses; analytic code; any other materials used in the review. | Page 26 |

*From:*  Page MJ, McKenzie JE, Bossuyt PM, Boutron I, Hoffmann TC, Mulrow CD, et al. The PRISMA 2020 statement: an updated guideline for reporting systematic reviews. BMJ 2021;372:n71. doi: 10.1136/bmj.n71. This work is licensed under CC BY 4.0. To view a copy of this license, visit <https://creativecommons.org/licenses/by/4.0/>
